# Modification of the head proteome of nurse honeybees (*Apis mellifera*) exposed to field-relevant doses of pesticides

**DOI:** 10.1038/s41598-020-59070-8

**Published:** 2020-02-10

**Authors:** Rodrigo Zaluski, Alis Correia Bittarello, José Cavalcante Souza Vieira, Camila Pereira Braga, Pedro de Magalhaes Padilha, Mileni da Silva Fernandes, Thaís de Souza Bovi, Ricardo de Oliveira Orsi

**Affiliations:** 1 Grupo de Estudos em Apicultura e Meliponicultura Sustentável de Mato Grosso do Sul - GEAMS, Federal University of Mato Grosso do Sul (UFMS), Faculty of Veterinary Medicine and Animal Science (FAMEZ), Campo Grande, MS Brazil; 2 São Paulo State University (UNESP), Institute of Biosciences, Department of Chemistry and Biochemistry, Botucatu, SP Brazil; 3 Federal University of Mato Grosso do Sul (UFMS), Institute of Chemistry (INQUI), Campo Grande, MS Brazil; 4 University of São Paulo (USP), Bauru Dental School, Bauru, SP Brazil; 5 Núcleo de Ensino, Ciência e Tecnologia em Apicultura Racional (NECTAR), São Paulo State University (UNESP), School of Veterinary Medicine and Animal Science, Department of Animal Production, Botucatu, SP Brazil

**Keywords:** Animal physiology, Entomology, Environmental impact, Conservation biology

## Abstract

Understanding the effect of pesticides on the survival of honeybee colonies is important because these pollinators are reportedly declining globally. In the present study, we examined the changes in the head proteome of nurse honeybees exposed to individual and combined pesticides (the fungicide pyraclostrobin and the insecticide fipronil) at field-relevant doses (850 and 2.5 ppb, respectively). The head proteomes of bees exposed to pesticides were compared with those of bees that were not exposed, and proteins with differences in expression were identified by mass spectrometry. The exposure of nurse bees to pesticides reduced the expression of four of the major royal jelly proteins (MRJP1, MRJP2, MRJP4, and MRJP5) and also several proteins associated with carbohydrate metabolism and energy synthesis, the antioxidant system, detoxification, biosynthesis, amino acid metabolism, transcription and translation, protein folding and binding, olfaction, and learning and memory. Overall, when pyraclostrobin and fipronil were combined, the changes in protein expression were exacerbated. Our results demonstrate that vital proteins and metabolic processes are impaired in nurse honeybees exposed to pesticides in doses close to those experienced by these insects in the field, increasing their susceptibility to stressors and affecting the nutrition and maintenance of both managed and natural colonies.

## Introduction

Pollination is an indispensable service provided mainly by wild insects and farmed honeybees (*Apis mellifera*, Linnaeus, 1758) that supports biodiversity, agriculture, and food security^1,2^. There is growing concern over the reported global reduction in these insects, which compromises this ecological service^2–8^.

The maintenance of honeybee colonies is dependent upon specialised tasks performed by individuals of different roles; thus, stress-related dysfunction of specialised workers can affect entire colonies^9,10^. Pesticides, phytochemicals, pathogens, and parasites are among the main stressors for honeybees, and exposure can lead to dysfunctions that change the behaviour, anatomy, and/or physiology of these insects^10^. Previous reviews have indicated that pesticide exposure negatively affects the maintenance of wild and commercial honeybee populations^5,11,12^. Exposure of bees to field-relevant doses of pesticides that are frequently found in plants visited by these insects compromise essential functions, such as cognition, foraging, navigation, homing, and memory^13–15^. Exposure also causes physiologic changes in individual bees that compromises colony maintenance^9,15–18^.

Nutrition is a key factor in colony maintenance^19^ and caste differentiation of honeybees, and is essential for the full development and activity of mandibular and hypopharyngeal (or brood-food) glands in the head of 6-day-old worker bees^20–22^. These glands are responsible for the production of royal jelly (RJ), a secretion used to nourish all bee larvae until 3 days old, after which only the queen larvae are fed with RJ throughout larval and adult life^21,23^. The development of brood food glands on nurse bees is associated with the consumption of high quantities of pollen that increases the exposure of these bees to the pesticides^24–27^.

Studies have demonstrated that, in colonies exposed to pesticides, queen production is reduced^16^, and an increase in queen supersedure occurs^17^. Generally, queens reared under these conditions have damaged ovarian tissues, high mortality, and worker rejection as well as difficulties with emerging, mating, and egg laying^28^. The low quality/quantity of RJ produced by nurse honeybees exposed to pesticides is a potential explanation for poor queen quality. In our previous study, we demonstrated that exposure of nurse honeybees to field-relevant doses of the systemic fungicide pyraclostrobin and insecticide fipronil caused morphological alterations in the mandibular and hypopharyngeal glands that can compromise RJ production and quality^15^.

Given previous findings, in the present study we investigated the changes in the proteome of honeybee heads that contain essential organs involved in the synthesis of RJ (mandibular and hypopharyngeal glands), sensorial structures (antennae), visual acuity (compound eyes and ocelli), and the brain, responsible for coordination and response to stimuli. Thus, it was possible to evaluate the occurrence of alterations in key proteins involved in the proper response of nurse bees to stimuli and the performance of their functions within the colony, and to detect changes in the metabolism of bees exposed to pesticides that consequently hamper colony maintenance. The proteomics analysis of the head of nurse honeybees chronically exposed to both pyraclostrobin and fipronil were conducted by protein fractionation (two-dimensional polyacrylamide gel electrophoresis (2D-PAGE)) and identification by electrospray ionisation-tandem mass spectrometry (ESI-MS/MS). The present study may contribute to a deeper understanding of the effects of chronic exposure of honeybees to pesticides and emphasises the need to reduce the use of these molecules and thus the exposure of bees.

## Results and Discussion

### Individual and combined effects of pyraclostrobin and fipronil on head proteome of nurse honeybees

The changes in the head proteome of nurse honeybees exposed to environmentally relevant doses of the fungicide pyraclostrobin, insecticide fipronil (850 and 2.5 ppb, respectively), and combinations of the two (pyraclostrobin + fipronil) were examined using the 2D-PAGE technique and compared with the proteome of bees not exposed to pesticides (control). Bees were exposed to pesticides in colonies where they received pollen patties contaminated for 6 d *ad libitum* (see Methods for details).

Image analysis of four colloidal Coomassie-stained 2D-PAGE gels for each treatment were analysed using Image Master 2-DE Platinum 7.0 software. Results showed 227 protein spots in the control group and 215, 221, and 211 spots in bees exposed to pyraclostrobin, fipronil, and pyraclostrobin + fipronil, respectively. A high scatter plot correlation coefficient was obtained between the four gels obtained in each treatment (98–100%). Protein spots were displayed within the isoelectric point (pI) range of 4.07–9.93 and mass range of 9.66–136.1 kDa. Gels from bees exposed to pesticides were individually subject to pairwise comparisons to the control group to deduce the fold expression level of proteins. For this, the relative volume parameter (%Vol) was used, which has been reported to be an efficient measure because it takes into account variation due to protein loading and staining by considering the total volume over all the spots in the gel^29–31^.

**Figure 1:**
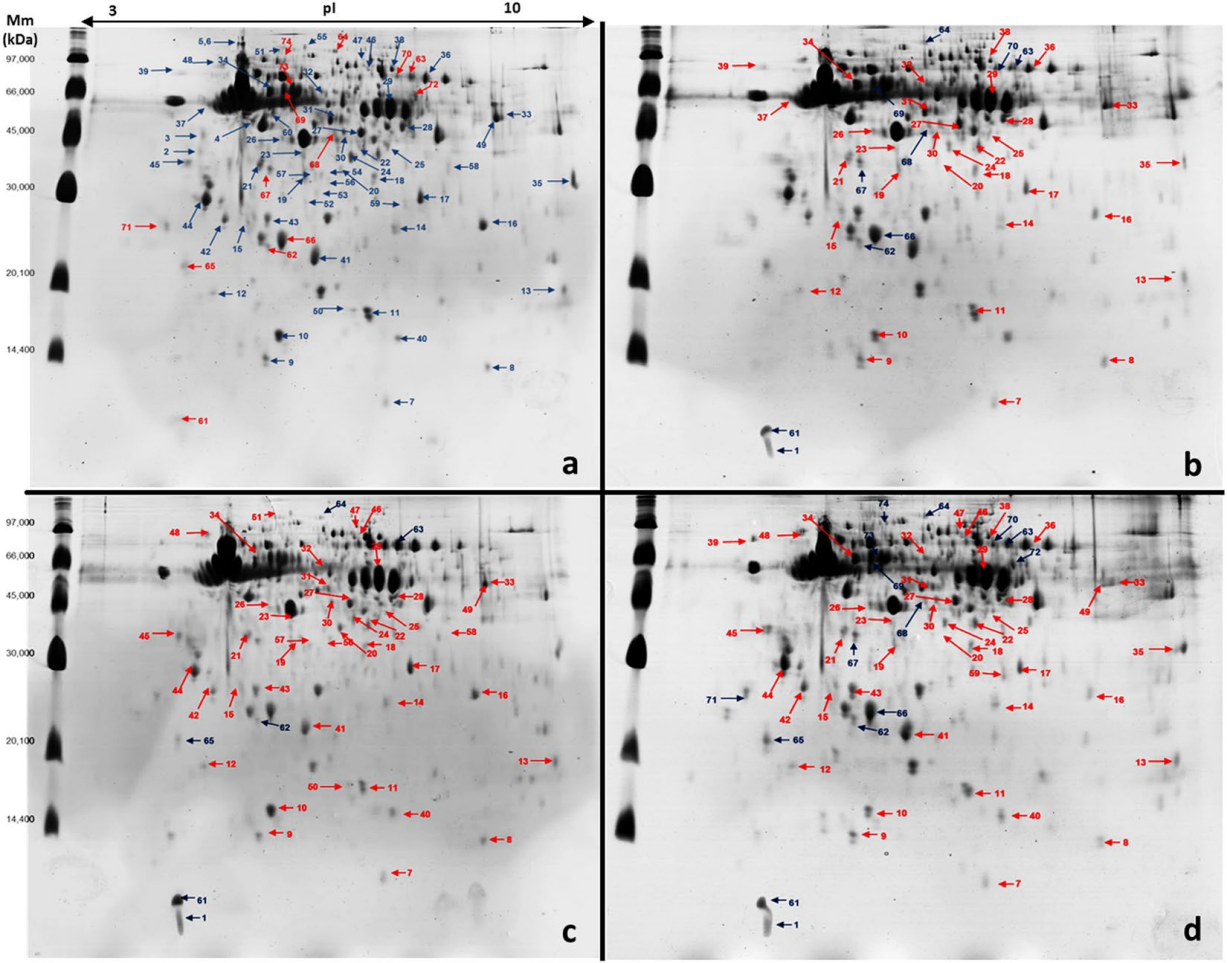
Representative 2D-PAGE gels from nurse honeybee heads (*Apis mellifera***)**. Bees exposed to field-relevant doses of pyraclostrobin and fipronil individually and combined. First dimension: 375 μg whole protein extracts from a pool of 30 honeybee heads on immobilised pH 3–10 nonlinear gradient strips (proteins were extracted in duplicate). Second dimension: 12.5% SDS-PAGE gels (two gels for each protein extract). Numbers represent ID of spots accepted as significantly differentially expressed between control and pesticide exposure treatments (see Supplementary Tables S1 and S2 for more details). Red spots represent decreased expression, and blue spots represent increased expression. (**a**) Control, (**b**) pyraclostrobin, (**c**) fipronil, and (**d**) pyraclostrobin + fipronil treatments.

When we compared protein expression between bees exposed to pyraclostrobin and the control group, 55 of the analysed spots were accepted as significantly differentially expressed between the two experimental conditions (10 were upregulated, 33 were downregulated, and 12 were uniquely expressed in the control group). In the analysis between bees exposed to fipronil and the control group, 56 of the analysed spots were differentially expressed (6 were upregulated, 44 were downregulated, and 6 were uniquely expressed in the control group). When we compare bees exposed to pyraclostrobin + fipronil and control group, 74 of the analysed spots were differentially expressed (15 were upregulated, 43 were downregulated, and 16 were uniquely expressed in the control group) (Fig. 1). Interestingly, in bees exposed to pyraclostrobin + fipronil, we observed higher fold-changes in the expression of spots that were up- and downregulated; additionally, the number of spots not present in bees exposed to this combination of pesticides was higher (Supplementary Tables S1 and S2).

All protein spots with significant changes in expression in bees exposed to pesticides and those found only in the control group were manually excised in gel digested with trypsin and analysed using ESI-MS/MS. In these analyses, 93 proteins were identified in spots downregulated in bees exposed to pesticides and 19 proteins in upregulated spots. Twenty-one proteins were identified in spots uniquely expressed in the control group, and 15 of these same proteins were also found in spots downregulated in bees exposed to pesticides. Only in four spots were the proteins unidentified due to their values being too low to produce a spectrum, or because the C.I.% of the database search was not higher than 95%, which was needed to yield unambiguous results.

Some proteins were identified in different protein spots possibly due to post-translational modifications that altered their molecular mass (Mm) and pI^32^, and more than one protein was identified in a single spot, as also observed in previous studies^31,33,34^. The identification of more than one protein in a single spot may be due to post-translational modifications^35^ or proteins belonging to multigenic families or isoforms, which generally have similar Mm and pI^36^.

Protein sequences were analysed using the Blast2GO tool to annotate with Gene Ontology (GO) terms and subsequent identification of the metabolic pathways in the Kyoto Encyclopedia of Genes and Genomes (KEGG), which allowed the separation of functional groups (Table 1).

**Table 1:** Biological functions and number of proteins identified in spots significantly differentially expressed in nurse honeybee (*Apis mellifera*) heads exposed to pyraclostrobin and fipronil individually and pyraclostrobin + fipronil.

	Pyraclostrobin		Fipronil		Pyraclostrobin + Fipronil	
Biological process/Function	Upregulated	Downregulated	Upregulated	Downregulated	Upregulated	Downregulated
Major royal jelly proteins	—	4	—	4	—	4
Carbohydrate metabolism and energy synthesis	2	11	1	11	3	11
Antioxidant system	—	12	—	16	—	18
Biosynthesis processes	—	5	—	7	—	7
Amino acid metabolism	—	4	—	5	—	5
Transcription/translation	1	4	—	5	1	6
Protein binding/folding	5	7	3	7	5	7
Olfactory system	—	1	—	1	—	1
Learning and memory	—	1	—	2	—	2
Stress response	3	—	3	—	3	—
Other/unknown functions	5	24	2	23	9	31
**Total**	**16**	**75**	**9**	**84**	**21**	**94**

Proteins spots were identified by ESI-MS/MS, and their sequences were analysed in Blast2GO to separate functional groups. Number of proteins up- and downregulated are presented based on the comparison of bees exposed to individual and combined effects of the fungicide and insecticide at field-relevant doses (850 and 2.5 ppb, respectively) and bees not exposed to pesticides (control).

**Figure 2:**
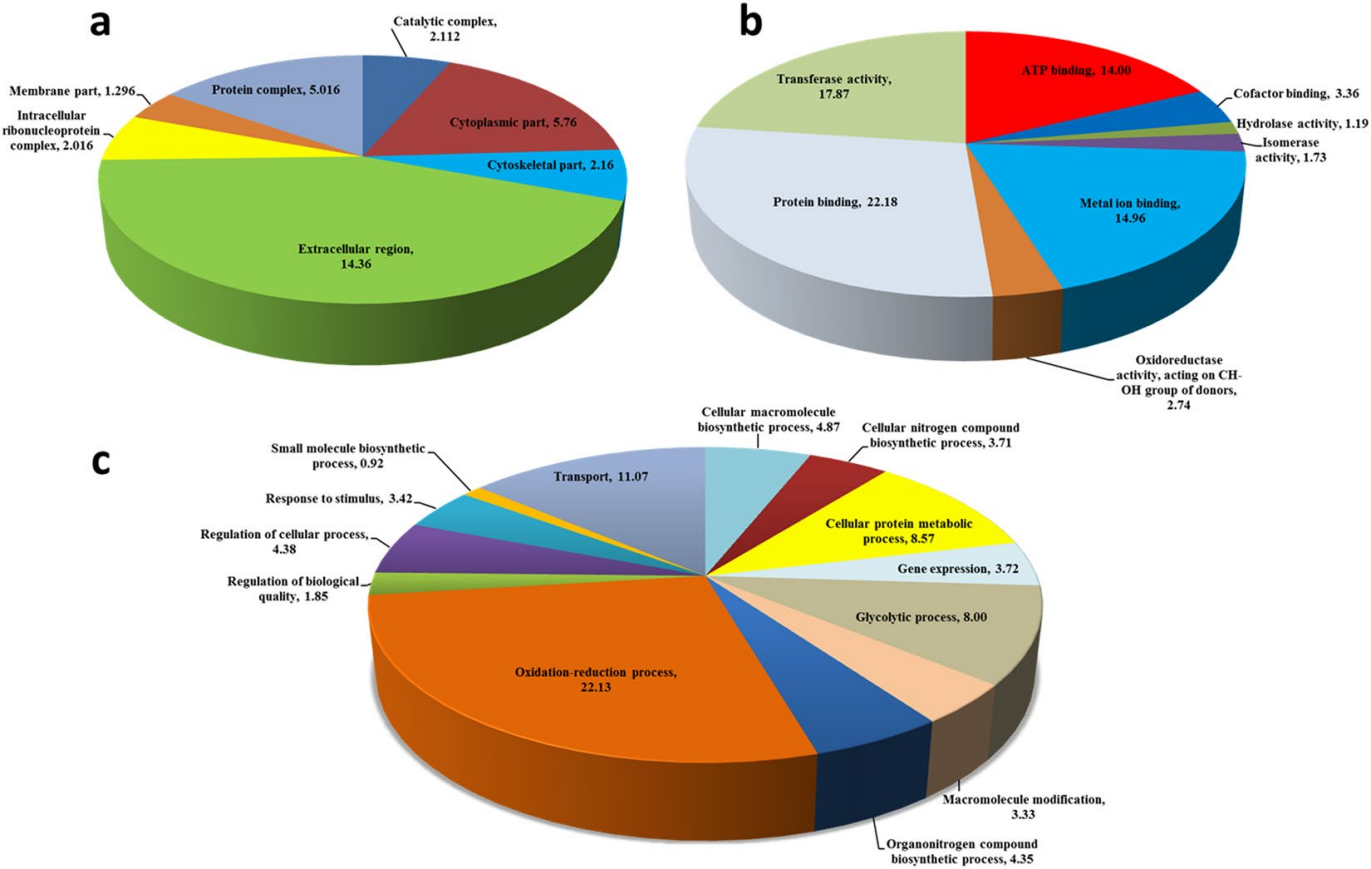
Classification of the protein sequences showing decreased expression (p < 0.05) in nurse bees (*Apis mellifera*) exposed to field-relevant doses of the fungicide pyraclostrobin and insecticide fipronil. (**a**) Cellular component, (**b**) molecular function, and (**c**) biologi**c**al process.

In general, the analysis of protein sequences identified in spots downregulated in bees exposed to pesticides in Blast2GO using GO resulted in scores mainly associated with the extracellular region, cytoplasm part, and protein complex and for the molecular functions of protein binding, transferase activity, metal ions binding, and ATP binding and for the biological processes of oxidation reduction, transport, cellular protein metabolism, and glycolysis (Fig. 2). Analysis of protein sequences identified in spots upregulated in bees exposed to pesticides resulted in scores mainly associated with the extracellular region, the molecular function of ATP binding, and various biological processes (Supplementary Fig. S1). These analyses demonstrated that exposure of nurse bees to pyraclostrobin and fipronil individually and in combination at field-relevant doses promoted changes in metabolism that may reduce the expression of proteins and increase their susceptibility to these molecules and other stressors, such as diseases, parasites, and other pesticides.

### Expression of major royal jelly proteins in nurse honeybees exposed to pesticides

The major RJ proteins (MRJPs) MRJP1, MRJP2, MRJP4, and MRJP5 were identified in spots that were downregulated in nurse bees exposed to pesticides or that were uniquely expressed in the control group. MRJP expression further decreased under treatment with pyraclostrobin + fipronil (Supplementary Table S1). Previous studies have reported that exposure of nurse honeybees to diets contaminated with pesticides caused morphological changes in hypopharyngeal glands^9,37,38^, and in both hypopharyngeal and mandibular glands^15^ that are responsible for the synthesis of RJ.

These studies suggested that morphologic alterations in these glands could quantitatively and qualitatively decrease the secretion of proteins in RJ. To our knowledge, our study is the first to demonstrate potential alterations in RJ protein produced by nurse honeybees exposed to pesticides. Nurse bees with impaired brood-food glands can potentially forage precociously, and this can reduce their life span^39^.

RJ proteins are key factors in colony nutrition, and antimicrobial defence^22,40^. Larvae destined to be queens receive only RJ during their development and adult life, whereas pollen and honey are added to the diet of worker-destined larvae^22^. The decrease in MRJP1 expression observed in our study is important because this protein has antimicrobial properties that are essential for larval health and decreasing susceptibility to pathogens^40,41^. Previous studies have reported impaired colony growth and queen production^16^, higher queen replacement rates^17^, decreased immunity^42^, and altered physiological development of queens^43^ that are reared in colonies exposed to pesticides. Our results suggest that these factors may be associated with changes in the quality of the RJ produced by nurse honeybees that consume pollen contaminated with pesticides. Future studies to evaluate the quality and quantity of RJ produced directly in colonies exposed to pesticides are necessary to confirm the effects observed in our study and to better understand the effects of pesticide exposure on larval nutrition, caste differentiation, and colony maintenance.

### General changes in metabolism of nurse honeybees exposed to pesticides

We identified important proteins involved in carbohydrate metabolism and energy synthesis (11 proteins), antioxidant system (18 proteins), and biosynthesis (seven proteins) in spots downregulated in nurse honeybees exposed to pesticides (Supplementary Table S1). These alterations characterise important changes in nurse honeybee metabolism. Once under normal conditions, nurse bees exhibited increased expression of proteins involved in these functions to perform their tasks in the colony^32^. Other important proteins that were downregulated were associated with amino acid metabolism, transcription/translation, protein binding/folding, olfactory system, learning and memory, and other proteins with other or unknown functions (Supplementary Table S1).

### Downregulation of enzymes involved in carbohydrate and energy metabolism: effect on ATP production

Reduction and/or absence of expression of the enzymes fructose-bisphosphate aldolase [Enzyme Commission (EC) number 4.1.2.13], triosephosphate isomerase (EC 5.3.1.1), phosphoglycerate mutase (EC 5.4.2.12), malate dehydrogenase (EC 1.1.1.37), aldose 1-epimerase (EC 5.1.3.3), glucose-6-phosphate isomerase (EC 5.3.1.9), and phosphomannomutase (EC 5.4.2.8) which participate in the processes of glycolysis and gluconeogenesis, and also the enzymes inorganic pyrophosphatase (EC 3.6.1.1), arginine kinase (EC 2.7.3.3), transaldolase (EC 2.2.1.2), and phosphoglycolate phosphatase-like (EC 3.1.3.18), which act in carbohydrate metabolism and feed into glycolysis pathway, characterise important changes in bees exposed to pesticides.

Decreased expression of these enzymes can impair ATP synthesis and lead to lower levels of the energy necessary to maintain protein biosynthesis, which may compromise RJ synthesis in nurse bees. Energy depletion may also impair detoxification processes that require high energy expenditure^44–46^. We also observed a decrease in the expression of arginine kinase, which is responsible for transporting ATP and is required for biochemical processes of the visual system, such as the regeneration of pigments in the retina^29^. According to Roat *et al*.^29^, who also observed decreased expression of this enzyme in bees exposed to fipronil, its reduction may impair vision in bees.

Our results corroborate those of previous studies that demonstrated impairment of energy metabolism in bees exposed to pesticides^47,48^. Suppression of ATP production by pyraclostrobin occurs by inhibiting the ubiquinol-oxidation centre of the mitochondria1 bc1 complex that blocks the electron transfer between cytochrome b and cytochrome c1 and disrupts ATP production^49^. Fipronil may impair ATP synthesis in bees due to the inhibition of the electron transport chain^50^. Mao *et al*.^48^ reported that bees exposed to fungicides did not metabolise some of the phytochemicals naturally present in pollen, and these molecules can compromise ATP synthesis. The expression of enzymes that act in glycolysis and gluconeogenesis is regulated, which limits their functions and activity aimed at meeting their metabolic rate^51^. Therefore, the inhibitory action of fipronil and pyraclostrobin on the electron transport chain and a possible influence of phytochemicals may have contributed to the decreased expression of enzymes involved in ATP synthesis. An additive effect, characterised by a further decrease in the expression of some enzymes involved in energy synthesis was observed in bees exposed to pyraclostrobin + fipronil. This result may be derived from the interaction between pesticides and nonmetabolised phytochemicals in pollen.

### Downregulation of antioxidant enzymes and increase in susceptibility to oxidative stress, pesticides, and diseases

We identified the following important enzymes: superoxide dismutase [Cu-Zn], peroxiredoxin 1, pyridoxine pyridoxamine 5-phosphate oxidase, 15-hydroxyprostaglandin dehydrogenase [NAD(+)]-like, lambda-crystallin homolog, trans-1,2-dihydrobenzene-1,2-diol dehydrogenase-like, glycerol-3-phosphate dehydrogenase [NAD(+)], malate dehydrogenase, aldose reductase-like, glycerol-3-phosphate dehydrogenase [NAD(+)], and probable medium-chain specific acyl-mitochondrial in downregulated spots in nurse bees exposed to pyraclostrobin + fipronil. Additionally, the enzymes glutathione S-transferase S1 and 3-hydroxyacyl- dehydrogenase type-2 were identified in downregulated spots in bees exposed to pyraclostrobin and pyraclostrobin + fipronil, and the enzymes 15-hydroxyprostaglandin dehydrogenase [NAD(+)]-like, Rab s geranylgeranyltransferase component A1, glutathione peroxidase, thioredoxin domain-containing 9, farnesol dehydrogenase-like, and prophenoloxidase (PO) were identified in downregulated spots in nurse bees exposed to fipronil and pyraclostrobin + fipronil.

All protein synthesis processes in eukaryotes demand high levels of ATP^52^, which consequently increases the production of reactive oxygen species (ROS)^32,53^. During ROS generation in cells, the expression of antioxidant enzymes increases to maintain homeostasis^46,54^. Our results demonstrated the downregulated antioxidant proteins in nurse bees exposed to pesticides that can increase their susceptibility to oxidative stress and were characterised by increased ROS generation and/or reduced physiological activity of antioxidant enzymes^55^. Some pesticides can act as pro-oxidants by impairing the functionality of antioxidants^56^ and to bees that presents a smaller number of genes that encode antioxidant proteins compared with the genomes of other insects^53^. This may represent an additional barrier to their defence against ROS^53,57^.

Higher susceptibility of nurse bees to oxidative stress may lead to damaged macromolecules^58^, DNA and RNA oxidation, lipid membrane peroxidation^59^, and impaired development of glands and decreased longevity^58^. Our previous study reported a reduction in the development of mandibular and hypopharyngeal glands in nurse honeybees exposed to pyraclostrobin and fipronil individually and in combination^15^. Other previous studies reported different responses of antioxidant system of honeybees exposed to pesticides or natural compounds, and these responses can change based on the age of the bee, time of exposure, dosage, and pesticide group^46,47,54,60,61^.

The enzyme superoxide dismutase [Cu-Zn] represents the first line of defence against ROS generated in mitochondria. This enzyme acts in the cytoplasm and converts superoxide radicals to oxygen and hydrogen peroxide, that are decomposed by catalases and peroxiredoxins, as peroxiredoxin 1^53^. An increase in superoxide dismutase isoforms^62^ and glutationes (GSTs) enzymes is associated with increased insect resistance to pesticides^63,64^. We found that these antioxidant enzymes were downregulated in bees exposed to fipronil, pyraclostrobin, and pyraclostrobin + fipronil that can increase the sensibility of these insect to these pesticides.

We also observed the downregulation of PO in bees exposed to fipronil without a synergic or addictive effect on their expression in bees exposed to combined pesticides, which suggests that fipronil is the main pesticide responsible for this effect. PO is an enzyme associated with immune response in bees and is involved in melanogenesis in invertebrates^65,66^, a key process in defence against bacteria, fungi, and viruses^65^. Thus, PO downregulation in bees exposed to fipronil can decrease the ability of bees to defend against microorganisms and increase their susceptibility to disease. A study by Zhu *et al*.^66^ reported that PO activity was reduced in bees exposed to pesticide mixtures.

### Downregulation of enzymes involved in biosynthesis and amino acid metabolism: implications for prostaglandin biosynthesis and detoxification

In nurse bees exposed to pyraclostrobin and fipronil we observed a decrease in the expression of the enzymes prostaglandin E synthase 3 (EC 5.3.99.3); pyridoxamine 5′-phosphate oxidase (EC 1.4.3.5); glutamine synthetase 2 cytoplasmic isoform X1 e glutamine synthetase 2 cytoplasmic (EC 6.3.1.2), involved in glutamine and arginine biosynthesis; 6-pyruvoyl tetrahydrobiopterin synthase (EC 4.2.3.12) involved in folate biosynthesis; alanine aminotransferase 1 (EC 2.6.1.2), involved in arginine biosynthesis; and hydroxyacylglutathione mitochondrial isoform X1 (EC 3.1.2.6) involved in glutathione biosynthesis.

Decreased expression of prostaglandin E synthase 3 can affect prostaglandin biosynthesis, which acts on reproduction, modulation of fluid secretion in glands, and in insect immune response^67^. The enzyme pyridoxamine 5′-phosphate oxidase is involved in the *de novo* synthesis of pyridoxine and pyridoxal phosphate, which are found in RJ and are essential to larval development of bees^19^.

The glutamine that is converted to glutamate is essential for glutathione biosynthesis and allows the functioning of enzymes in the class glutathiones (GSTs), the synthesis of polyamines that interact with DNA/RNA, and protein biosynthesis^54^. The downregulation of proteins that are involved in glutamine and glutathione biosynthesis reflects the decreased expression of GST enzymes observed in bees exposed to fipronil, pyraclostrobin, and pyraclostrobin + fipronil, indicating an indirect reduction in their detoxification ability. Additionally, the downregulation of protein-L-isoaspartate O-methyltransferase (EC 2.1.1.77) and s-methyl-5′-thioadenosine phosphorylase (EC 2.4.2.28) involved in cysteine and methionine metabolism, and alanine aminotransferase 1 (EC 2.6.1.2), glutamine synthetase 2 cytoplasmic isoform X1, and glutamine synthetase 2 cytoplasmic (EC 6.3.1.2) involved in alanine, aspartate, and glutamate metabolism (except alanine aminotransferase 1 to pyraclostrobin) also support the hypothesis of decreased detoxification ability in bees exposed to fipronil, pyraclostrobin, and pyraclostrobin + fipronil. Amino acid catabolism contributes to energy generation and can generate Krebs cycle intermediates used as precursors for the synthesis of molecules such as glutathione that are involved in detoxification^54^.

### Downregulation of enzymes involved in transcription/translation

The proteins ubiquitin-60S ribosomal L40, eukaryotic translation initiation factor 3 subunit I, elongation factor 1-gamma, and tyrosine–tRNA ligase were identified in downregulated spots in bees exposed to fipronil, pyraclostrobin, and pyraclostrobin + fipronil. The enzyme U3 small nucleolar RNA-associated 4 homolog A was identified in spots that presented downregulation in bees exposed to fipronil and to association of pesticides, and the protein eukaryotic initiation factor 4A-I was identified in spots downregulated in bees exposed to association of pesticides. These proteins have functions involved in RNA translation and/or transport processes^68^ and their downregulation suggests a reduction of protein synthesis in bees exposed to pesticides.

### Downregulation of enzymes involved in protein binding/folding

The proteins hsp70-binding 1, 10 kda heat shock mitochondrial, profilin, t-complex 1 subunit eta, and 60 kda heat shock mitochondrial-like have protein binding functions, such as chaperone activity or in protein folding processes that are essential for maintaining the functionality of proteins, and their expression decreased in all groups exposed to pesticides. These results suggest that proteins with essential functions pertaining to the conformation and adequate functioning of other proteins can be affected by the exposure of bees to pesticides. Proline binds to actin and has important structural functions. The 60 kda heat shock mitochondrial-like 6 protein is involved in protein folding and processing of genetic information and RNA, guaranteeing the turnover of RNA molecules, a process that is critical for gene expression^68^.

### Downregulation of proteins involved in olfaction, learning, and memory

The expression of the protein odorant-binding protein 14 (OBP14) was decreased in all bees exposed to pesticides. The odorant proteins are required for the adequate recognition of chemical stimuli by the insect olfactory system^69^, and decreased expression can impair the perception of stimuli by nurse bees and affect their functionality in the colony. The proteins 14-3-3 epsilon and 14-3-3 zeta that are involved in learning and memory in insects^70,71^ were also downregulated in bees exposed to pesticides. These results suggest an impairment of these functions in bees due to exposure to fipronil, pyraclostrobin, and pyraclostrobin + fipronil.

### Proteins upregulated in bees exposed to pesticides

Some proteins involved in carbohydrate metabolism and energy synthesis [malic enzyme (EC 1.1.1.40), pyruvate dehydrogenase E1 component subunit mitochondrial (EC 1.2.4.1), and pyruvate kinase (EC 2.7.1.40)], others involved in protein synthesis (eukaryotic translation initiation factor 3 subunit K), and some proteins with other or unknown functions were identified in spots upregulated in bees exposed to pesticides (Supplementary Table S2).

We observed an increase in the expression of heat shock 70 kda cognate 5, heat shock 70 kda 4 isoform X1, and heat shock 70 kda cognate 3, which are in the heat shock protein (HSP) family. These proteins are cellular markers of stress response, and an increase in these proteins in *Drosophila melanogaster* and bees exposed to pesticides has been previously reported^37,72^. HSPs can prevent cell apoptosis^73,74^ and protein denaturation and can promote the degradation of abnormal proteins^73^; however, the effects of their upregulation remains poorly understood. In addition, two isoforms of the protein lethal(2)essential for life–like, which are also in the HSP family and function as chaperones exhibited increased expression in bees exposed to pesticides. A study performed by Roat *et al*.^29^ reported increased expression of lethal(2)essential for life–like in bees exposed to fipronil. We suggest that increased expression of HSP proteins and chaperones can be involved in the cytotoxic effects of fipronil and increase the damage to protein structure caused by oxidative stress in bees exposed to pesticides.

Our results demonstrated that exposure of nurse honeybees to field-relevant doses of the pyraclostrobin, fipronil, and pyraclostrobin + fipronil caused changes in vital metabolic processes, and we characterised new effects associated with exposure of bees to pesticides. These changes interfered with nurse bee functions that are essential for the development and maintenance of their colony, increasing the susceptibility of the colony to collapse. Nurse bees with impaired brood-food glands^15^ and lower capacity of RJ production can potentially forage precociously, and this can reduce their life time^39^.

The global analysis of protein profiles in nurse honeybee heads with the 2D-PAGE technique allowed us to screen the main changes that occur when bees are exposed to pesticides. However, our results also highlight the necessity for further detailed research to examine changes in protein expression and to evaluate the effects of pesticides on pollinators to detect chronic effects that may compromise colony maintenance and to fully understand the effects of pesticides on honeybee colony functionality. Owing to the negative effects of exposure to pyraclostrobin and fipronil observed in our study, it seems evident that pesticide use for the maintenance of native and managed pollinators should be reduced.

## Methods

### Experimental design, diet supply, residue analysis, and nurse bee collection

Test subjects were Africanized honeybees kept in the apiary of the Faculty of Veterinary Medicine and Animal Science, São Paulo State University, Botucatu, São Paulo, Brazil, under normal living conditions. All colonies were free of disease and parasites; thus, no treatment against *Varroa destructor* was necessary. All procedures to obtain the nurse bees, expose them to pesticides, and confirm the presence of pesticides in the pollen patties supplied to the colonies followed those detailed in our previous study (Zaluski *et al*. 2017). Briefly, approximately 40 newly emerged bees (<10 h) of five donor colonies were marked with a specific colour using a non-toxic paint (Posca Paint Pens, Mitsubishi Pencil, Japan) to distinguish colony origin and were randomly distributed in experimental colonies where they received contaminated food. Each experimental colony was housed in a nucleus with two sealed brood frames, two open brood frames, one egg-laying frame, and approximately 4,000 bees. The queens in each experimental nucleus were sisters, naturally mated and at four months of age. All frames with pollen/bee bread were removed from the experimental colonies 24 h before marked bees were introduced, and pollen traps were installed at their entrances to maximise the amount of contaminated diet consumed. We highlight that our experimental design considered the nurse honeybees as experimental units to ensure the genetic diversity of the honeybees, lower variation in the development of brood food glands, and that all tested bees came from the same donor colonies^15^. This technique avoided potential colony-specific changes (e.g. nutrition and temperature changes) that may interfere with the development of glands during the larval and pupal phases^21^, and consequently produce changes not related to pesticide exposure during the nursing phase.

Stock solutions of pyraclostrobin and fipronil were prepared from formulated products (Comet 250 g a.i. L^−1^, inert ingredients 802 g L^−1^, BASF Schwarzheide GmbH, Schwarzheide, Germany; Regent 800WG 800 g a.i. kg^−1^, inert ingredients 200 g kg^−1^ BASF Agri-Production SAS, Saint Aubin Les Elbeuf, France) diluted in distilled water. The final concentration of each treatment dose (pyraclostrobin: 850 ppb; fipronil: 2.5 ppb; pyraclostrobin + fipronil: 850 ppb + 2.5 ppb) was obtained by adding concentrated pyraclostrobin and fipronil solutions to honey syrup (50% w/v), which was then added to pollen powder to a 3:1 (pollen:honey syrup) ratio. Pollen patties were homogenised, portioned in cellophane paper (100 g), and stored in a freezer (−20 °C) until use. Control pollen patties without pesticides were prepared following the same procedure without the addition of pesticides.

The residue of pollen patties was analysed by the QuEChERS (quick, easy, cheap, effective, rugged, and safe) pesticide extraction method and ultra-high-performance liquid chromatography coupled with tandem mass spectrometry (UHPLC-MS/MS)^75^, which confirmed the absence of pesticides in the control patties. Residue level in patties containing pyraclostrobin was 870 ± 53 ppb (mean ± SD); however, none of the fipronil residues or metabolites (fipronil sulfone, fipronil desulfinyl, fipronil sulfide, or fipronil amide) were detected in our contaminated patties within the limit of detection (1.5 ppb). Given the clear biological changes that we observed in our morphologic study^15^, and in the present report, fipronil was likely present in pollen patties and had an effect even in concentrations below the limit of detection. The concentrations of pesticides used in our study enabled us to expose the bees to diets containing pyraclostrobin^76–78^ and fipronil^79–81^ in conditions similar to those occurring naturally.

Each experimental colony received one contaminated pollen patty daily (for 6 d), and marked honeybees consumed contaminated pollen *ad libitum* from the first day until nursing when they produce more RJ^21,32,82,83^. For proteomic analysis, 60 marked nurse honeybees (12 per donor colony, distinguished according to their colour) were collected in each experimental colony on day 7 (after 6 d of exposure to the contaminated diet). Bees were decapitated and the samples stored at −80 °C until the proteomic analyses were performed.

### Sample preparation and electrophoretic runs (2D-PAGE)

Thirty bee heads from each treatment (six bees per donor colony discerned based on specific colours used in their marking) were washed with cold acetic acid (1%) and double rinsed with ultrapure water to remove impurities. Proteins were extracted in duplicate (two pools of 30 bee heads) with 1 mL of ultrapure water, using an OMNI Bead Ruptor and 2.8 mm ceramic beads (OMNI International, Kennesaw, GA, USA) (speed = 5, cycles = 2, time = 60 s); between cycles, samples were kept in ice for 5 min. The extraction process using ultrapure water was used to maximise the extraction of water-soluble proteins, which include proteins from RJ^84^ and antioxidant enzymes. The protein extracts were separated from the solid portion by centrifugation at 8,000 *g* for 15 min at 4 °C. Subsequently, the supernatant was filtered through a sterile syringe filter (0.46 μm), precipitated with cold acetone (80%) at a 1:4 ratio (sample:acetone) and kept at 10 °C for 2 h for protein precipitation. Next, the protein precipitate was centrifuged at 8,000 *g* for 10 min at 4 °C, the supernatant was removed, and the protein pellets were washed twice more with the ice-cold acetone.

Part of the protein pellets obtained under each treatment were solubilised in 0.50 mol L^−1^ NaOH and used to measure total protein concentration in the head samples using the Quick Start Bradford Protein Assay Kit (Bio-Rad, Hercules, CA, USA) and bovine serum albumin as the standard. Absorbance readings were performed in a spectrophotometer (Spectrophotometer Evolution 60, Thermo Fisher Scientific, Waltham, MA, USA) at a wavelength of 595 nm.

The electrophoretic runs were performed using head samples for the different experimental groups (bees exposed to pyraclostrobin, fipronil, pyraclostrobin + fipronil, and the control). Four gels were made for each group (two for each protein extract obtained from a pool of 30 bee heads). Protein pellets were diluted in a solution containing 7 mol L^−1^ urea, 2 mol L^−1^ thiourea, 2% (w/v) CHAPS (3-[(3-cholamidopropyl)-dimethylammonio]-1-propanesulfonate), 0.5% (v/v) ampholytes at pH 3−10, 0.002% (w/v) bromophenol blue, and 2.8 mg of dithiothreitol (DTT). Subsequently, a total of 375 μg of protein (diluted in 250 μL of buffer) was added to 13-cm strips containing polyacrylamide gel with ampholytes immobilised at pH 3–10. These strips were placed onto the first dimension focusing for 12 h at 25 °C to be rehydrated with the protein extract. After this period, the strips were placed into an Ettan IPGphor isoelectric focusing unit (GE Healthcare, Chicago, IL, USA) for the first-dimension separation, totalling 15,504 Vh. After, the strips were reduced for 15 min with a solution containing 6 mol L^−1^ urea, 2% (w/v) SDS, 30% (v/v) glycerol, 50 mmol L^−1^ Tris-HCl, 0.002% (w/v) bromophenol blue, and 2% (w/v) DTT and alkylated for 15 min with a similar solution but with DTT replaced by 2.5% (w/v) iodoacetamide.

These procedures enabled the alkylation of the thiol groups of the proteins and prevented their reoxidation^31^. To resolve the second dimension, the strips were applied to 12.5% polyacrylamide gels. A piece of filter paper with 10 μL of protein molecular weight markers (14.4–97.0 kDa range) was placed close to the strip in the gel, and they were sealed with a hot solution of 0.5% (m/v) agarose and run in an SE 600 Ruby electrophoresis unit (GE Healthcare) with two steps: 7.5 mA/gel for 30 min and 15 mA/gel for 2 h 10 min.

After the first and second dimensions in the electrophoretic runs, the proteins were fixed in the gels using a solution containing 10% (v/v) acetic acid and 40% (v/v) ethanol for 1 h. In the sequence, the proteins were revealed with a colloidal Coomassie stain: 8% (w/v) ammonium sulfate, 1.6% (v/v) phosphoric acid, 0.08% (w/v) Coomassie Brilliant Blue G-250, and 25% (v/v) methanol for 72 h. Afterwards, the Coomassie stain was removed and the gels were washed with ultrapure water. The gels were scanned using ImageScanner III (GE Healthcare) at 300 dpi resolution and 100% zoom. The images were analysed by ImageMaster 2D Platinum 7.0 (GE Healthcare Life Sciences) to obtain the number of spots, percentage of correlation between gels (matching), pI, volume, and Mm. The parameters used for automatic detection of spots were: protrusion: 20, smooth: 03, and minimum area: 05. The contrast of the gel images was adjusted to allow better visualisation of the spots, and images were manually edited when necessary.

### Analysis of the differences in expression of proteins

Gels from bees exposed to all pesticides were individually subject to pairwise comparisons to the control group to deduce fold expression level on protein spots using the relative volume parameter (%Vol) of the total spots per group^29–31^. For differential expression analysis, statistical significance was estimated by one-way ANOVA performed in ImageMaster 2D Platinum 7.0. Spots displaying statistically significant differential expression (α = 0.05) were selected for protein identification.

### Protein identification by ESI-MS/MS and bioinformatics analyses

Proteins spots were excised from the gels and digested with trypsin as described previously^85^. Aliquots of solutions containing peptides were analysed by obtaining the mass spectra using the nanoACQUITY UPLC-Xevo QT-MS (Waters, Wilmslow, UK) system with ESI. Data were acquired over 20 min, and the scan range was 50–2000 Da. ProteinLynx Global Server version 3.0 was used to process and search the continuum LC–MS^E^ data, setting carbamidomethylation of cysteines as the fixed modification and oxidation of methionines as the variable modification, allowing one missing cleavage and a maximum error tolerance of 10 ppm^31^. Proteins were identified using the UniProt database (UniProtKB/Swiss-Prot − www.uniprot.org), and the search was conducted for *A. mellifera*. Fasta sequences of proteins were obtained in UniProtKB/Swiss-Prot database and imported to Blast2GO software to annotate the proteins by function using the sequence similarity based on GO and its separation at three levels (cellular component, molecular function, and biological function)^86^. Metabolic pathways were searched for in the KEGG database with indication of the EC number to demonstrate the chemical reactions catalysed by the enzymes identified^68^.

## Supplementary information

Supplementary Information 1.

Supplementary Information 2.

## Data Availability

The datasets generated and/or analysed in the current study are available from the corresponding author on reasonable request.
