# Author Correction: Modification of the head proteome of nurse honeybees (*Apis mellifera*) exposed to field-relevant doses of pesticides

**DOI:** 10.1038/s41598-020-63289-w

**Published:** 2020-04-07

**Authors:** Rodrigo Zaluski, Alis Correia Bittarello, José Cavalcante Souza Vieira, Camila Pereira Braga, Pedro de Magalhaes Padilha, Mileni da Silva Fernandes, Thaís de Souza Bovi, Ricardo de Oliveira Orsi

**Affiliations:** 1 Grupo de Estudos em Apicultura e Meliponicultura Sustentável de Mato Grosso do Sul - GEAMS, Federal University of Mato Grosso do Sul (UFMS), Faculty of Veterinary Medicine and Animal Science (FAMEZ), Campo Grande, MS Brazil; 2 São Paulo State University (UNESP), Institute of Biosciences, Department of Chemistry and Biochemistry, Botucatu, SP Brazil; 3 Federal University of Mato Grosso do Sul (UFMS), Institute of Chemistry (INQUI), Campo Grande, MS Brazil; 4 University of São Paulo (USP), Bauru Dental School, Bauru, SP Brazil; 5 Núcleo de Ensino, Ciência e Tecnologia em Apicultura Racional (NECTAR), São Paulo State University (UNESP), School of Veterinary Medicine and Animal Science, Department of Animal Production, Botucatu, SP Brazil

Correction to: *Scientific Reports* 10.1038/s41598-020-59070-8, published online 10 February 2020

The Acknowledgements section in this Article is incomplete.

“This study was supported by the Brazilian Council for Scientific and Technological Development/CNPq (grant number: 165696/2014-1). The authors thank the Pro Reitoria de Pós Graduação, Univ. Estadual Paulista (UNESP), from São Paulo state, Brazil for the financial support.”

should read:

“This study was financed in part by the Coordenação de Aperfeiçoamento de Pessoal de Nível Superior – Brasil (CAPES) – Finance Code 001. The authors gratefully acknowledge financial support from the Brazilian Council for Scientific and Technological Development/CNPq (grant number: 165696/2014-1), and the Universidade Federal de Mato Grosso do Sul.”

