# Supplementary material for: Modification of the head proteome of nurse honeybees (Apis mellifera) exposed to field-relevant doses of pesticides
Source: Sci Rep. 2020 Feb 10;10:2190. doi: 10.1038/s41598-020-59070-8 (PMC7010795; doi:10.1038/s41598-020-59070-8)
Supplement: Supplementary file 1 — Supplementary Information 1. [file 41598_2020_59070_MOESM1_ESM.docx]

**Supplementary Figure S1: Modification of the head proteome of nurse honeybees (*Apis mellifera*)** **exposed to field-relevant doses of pesticides**

**Rodrigo Zaluski, Alis Correia Bittarello, José Cavalcante Souza Vieira, Camila Pereira Braga, Pedro de Magalhaes Padilha, Mileni da Silva Fernandes, Thaís de Souza Bovi, Ricardo de Oliveira Orsi**


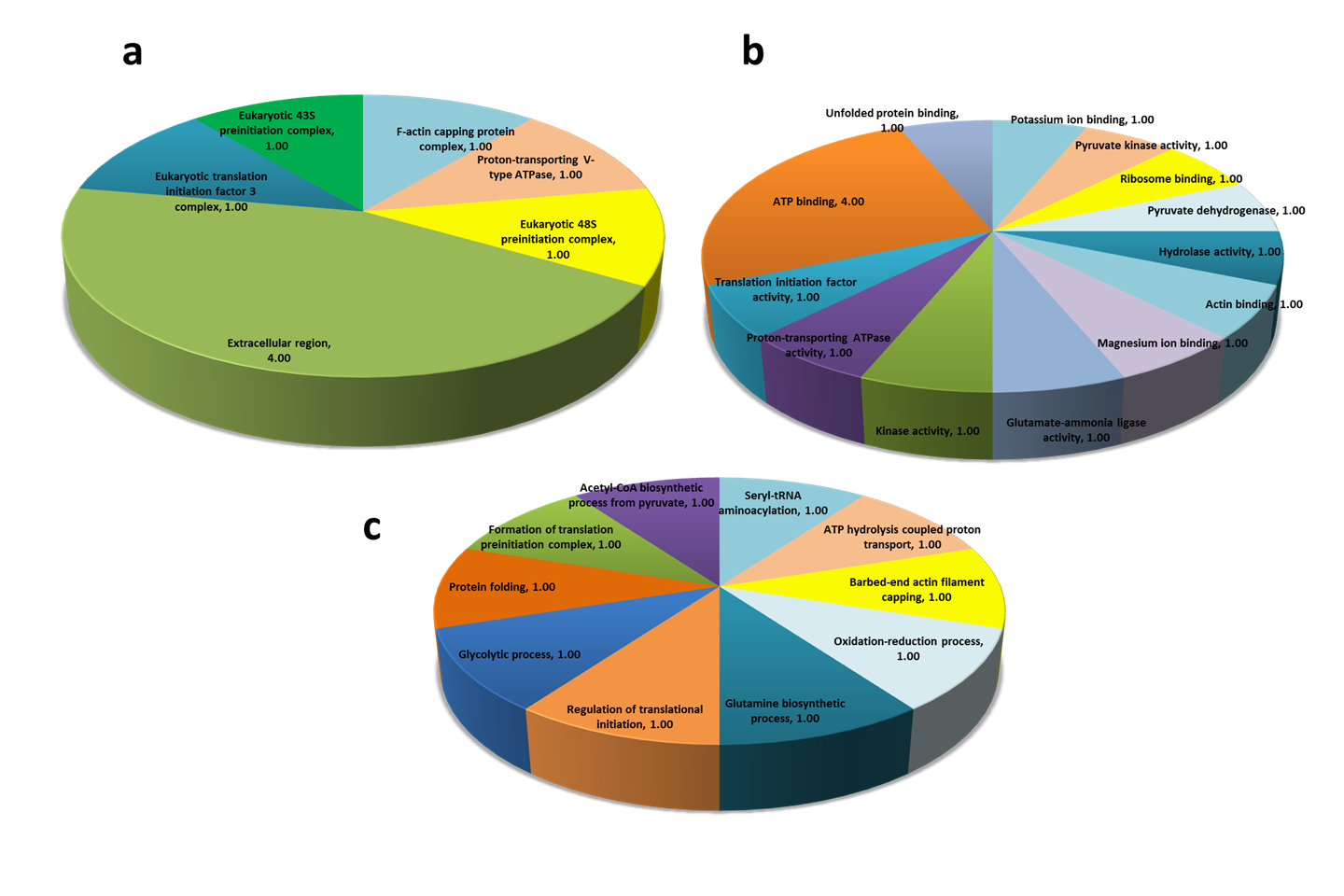


**Supplementary Fig. S1.** Classification of the protein sequences showing increased expression (α= 0.05) in nurse bees (*Apis mellifera*) exposed to field-relevant doses of the fungicide pyraclostrobin and insecticide fipronil. (**a**) Cellular component, (**b**) molecular function, and (**c**) biological process.
