# Supplementary material for: Modification of the head proteome of nurse honeybees (Apis mellifera) exposed to field-relevant doses of pesticides
Source: Sci Rep. 2020 Feb 10;10:2190. doi: 10.1038/s41598-020-59070-8 (PMC7010795; doi:10.1038/s41598-020-59070-8)
Supplement: Supplementary file 2 — Supplementary Information 2. [file 41598_2020_59070_MOESM2_ESM.docx]

**Supplementary Tables: Modification of the head proteome of nurse honeybees (*Apis mellifera*)** **exposed to field-relevant doses of pesticides**

**Rodrigo Zaluski, Alis Correia Bittarello, José Cavalcante Souza Vieira, Camila Pereira Braga, Pedro de Magalhaes Padilha, Mileni da Silva Fernandes, Thaís de Souza Bovi, Ricardo de Oliveira Orsi**

**Supplementary Table S1 ……………………………………………………………………..…………………………………….……….Page 02**

**Supplementary Table S2 ………………………………………………………………………………………………………….………...Page 11**

**Supplementary Table S1. List of proteins identified in spots downregulated (ANOVA; α= 0.05) of nurse honeybees exposed to pyraclostrobin (P), fipronil (F) and pyraclostrobin + fipronil (PF).** The expression analysis was performed with Image Master Platinum software, using as a reference the proteome of bees not exposed to pesticides (control), and the protein identification was performed by ESI-MS/MS. A negative signal (-) indicated potential reduction of protein expression in bees exposed to pesticides. The signal Ø indicated absence of protein spots in the groups exposed to pesticides. In this case, the proteins were identified in the control group. ^a^Spot ID numbers refer to the numbers on the 2D-PAGE gels shown in Fig. 1. ^b,c^Protein accession number and score according to the MASCOT software. ^d^Experimental pI and Mw of proteins obtained in Platinum Image Master software from gel analyses. ^e^Theoretical pI and Mw of proteins obtained in UniProtKB/Swiss-Prot database. ^f^Protein coverage indicates the percentage of residues from the sequences that were identified by the spectrometer for each protein. ^g^Role in biological process according UniProtKB-KW database. Proteins that have more than one function are presented repeatedly.

| Spot ID^a^ | Fold  Change in P | Fold  Change in F | Fold  Change in PF | Protein | Access^b^ | Score^c^ | pI/MM experimental^d^ | pI/MM theoretical^e^ | Coverage (%)^f^ | Molecular function^g^ |
| --- | --- | --- | --- | --- | --- | --- | --- | --- | --- | --- |
| **Major Royal Jelly Proteins (MRJPs)** | | | | | | | | | | |
| 02 | Ø | Ø | Ø | Major royal jelly protein 1 | O18330 | 4673.20 | 4.63/41.157 | 5.10/48.886 | 33.56 | caste determination, influence by environmental factors; defense response to fungus; defense response to Gram˗negative bacterium; defense response to Gram˗positive bacterium; killing of cells of other organism |
| 03 | Ø | Ø | Ø |  |  | 7974.65 | 4.74/48.757 |  | 45.14 |  |
| 04 | Ø | Ø | Ø |  |  | 1009.55 | 5.36/50.976 |  | 10.65 |  |
| 05 | Ø | Ø | Ø |  |  | 12723.76 | 5.24/133.690 |  | 58.56 |  |
| 06 | Ø | Ø | Ø |  |  | 8356.32 | 5.17/136.100 |  | 48.84 |  |
| 15 | ˗1.68 | ˗1.78 | ˗2.19 |  |  | 3615.26 | 5.39/26.334 |  | 32.41 |  |
| 24 | ˗2.40 | ˗1.45 | ˗2.50 |  |  | 1320.00 | 6.77/39.214 |  | 42.36 |  |
| 28 | ˗1.98 | ˗2.27 | ˗2.54 |  |  | 2033.81 | 7.27/45.000 |  | 44.68 |  |
| 32 | ˗1.33 | ˗1.08 | ˗1.73 |  |  | 4900.29 | 6.54/57.427 |  | 58.8 |  |
| 33 | ˗1.60 | 1.85 | ˗2.47 |  |  | 6943.43 | 8.46/63.974 |  | 55.56 |  |
| 34 | ˗1.15 | ˗1.14 | ˗1.70 |  |  | 12725.02 | 5.60/66.472 |  | 79.63 |  |
| 36 | ˗1.09 | – | ˗1.56 |  |  | 1296.31 | 6.95/56.980 |  | 50.23 |  |
| 49 | – | ˗1.07 | ˗2.05 |  |  | 6710.26 | 8.26/59.726 |  | 54.86 |  |
| 60 | – | – | Ø |  |  | 9309.71 | 5.92/56.223 |  | 69.21 |  |
| 12 | ˗1.00 | ˗1.01 | ˗1.15 | Major royal jelly protein 2 | O77061 | 466.72 | 4.90/18.909 | 6.83/51.074 | 33.41 | – |
| 18 | ˗1.74 | ˗1.74 | ˗2.00 |  |  | 1980.23 | 7.12/33.170 |  | 30.53 | – |
| 29 | ˗1.28 | ˗2.29 | ˗2.57 |  |  | 13689.63 | 7.29/45.000 |  | 50.22 | – |
| 36 | ˗1.09 | – | ˗1.56 |  |  | 17707.96 | 6.95/56.980 |  | 76.55 | – |
| 37 | ˗1.11 | – | Ø |  |  | 1295.67 | 4.75/57.746 |  | 23.89 | – |
| 44 | – | ˗1.17 | ˗1.46 |  |  | 4160.62 | 4.74/29.021 |  | 35.40 | – |
| 49 | – | ˗1.07 | ˗2.05 |  |  | 6554.41 | 8.26/59.726 |  | 55.75 | – |
| 33 | ˗1.60 | ˗1.85 | ˗2.47 | Major royal jelly protein 2 | A0A088AU22 | 1766.85 | 8.46/63.974 | 8.07/47.553 | 14.53 | – |
| 29 | ˗1.28 | ˗2.29 | ˗2.57 | Major royal jelly protein 2 | A0A088AU25 | 11164.63 | 7.29/45.000 | 5.67/37.105 | 44.79 | – |
| 36 | ˗1.09 | – | ˗1.56 |  |  | 13279.21 | 6.95/56.980 |  | 68.10 | – |
| 29 | ˗1.28 | ˗2.29 | ˗2.57 | Major royal jelly protein 2 | A0A088AU26 | 13798.74 | 7.29/45.000 | 6.60/ 53.797 | 52.73 | – |
| 44 | – | ˗1.17 | ˗1.46 |  |  | 4125.76 | 4.74/29.021 |  | 30.67 | – |
| 36 | ˗1.09 | – | ˗1.56 |  |  | 17442.77 | 6.95/56.980 |  | 77.73 | – |
| 37 | ˗1.11 | – | Ø | Major royal jelly protein 2 | A0A088AU27 | 8466.33 | 4.75/57.746 | 4.91/ 50.851 | 57.53 | – |
| 21 | ˗1.80 | ˗1.73 | ˗2.22 | Major royal jelly protein 4 | Q17061 | 2095.66 | 5.50/37.782 | 5.89/ 52.915 | 24.35 | – |
| 34 | ˗1.15 | ˗1.14 | ˗1.70 |  |  | 2367.30 | 5.60/66.472 |  | 36.21 | – |
| 60 | – | – | Ø |  |  | 458.67 | 5.92/56.223 |  | 9.05 | – |
| 03 | – | – | Ø | Major royal jelly protein 5 | O97432 | 1185.53 | 4.74/48.757 | 5.95/ 70.236 | 37.88 | – |
| 12 | ˗1.00 | ˗1.01 | ˗1.15 |  |  | 285.40 | 4.90/18.909 |  | 34.88 | – |
| 32 | ˗1.33 | ˗1.08 | ˗1.73 |  |  | 806.93 | 6.54/57.427 |  | 22.07 | – |
| 33 | ˗1.60 | ˗1.85 | ˗2.47 |  |  | 1766.85 | 8.46/63.974 |  | 13.18 | – |
| **Carbohydrate metabolism and energy synthesis** | | | | | | | | | | |
| 03 | Ø | Ø | Ø | Fructose˗bisphosphate aldolase | A0A087ZPQ8 | 1087.87 | 4.74/48.757 | 8.33/41.713 | 40.31 | fructose˗bisphosphate aldolase activity |
| 16 | ˗2.46 | ˗1.58 | ˗2.65 | Triosephosphate isomerase | A0A088ARS9 | 34491.68 | 8.68/28.711 | 7.84/13.702 | 88.98 | gluconeogenesis; glycolytic process; pentose–phosphate shunt |
| 58 | Ø | ˗1.20 | Ø |  |  | 913.24 | 8.19/37.224 |  | 68.5 |  |
| 19 | ˗1.16 | ˗1.43 | ˗1.63 | Arginine kinase | O61367 | 536.64 | 6.12/34.233 | 5.66/40.008 | 33.24 | arginine kinase activity; ATP binding |
| 55 | Ø | – | Ø |  |  | 340.07 |  |  | 19.44 |  |
| 21 | ˗1.80 | ˗1.73 | ˗2.22 | Phosphoglycolate phosphatase˗like | A0A087ZPT8 | 40227.95 | 5.50/37.782 | 5.15/34.139 | 83.39 | phosphatase activity; hydrolase |
| 02 | Ø | Ø | Ø |  |  | 3302.60 | 4.63/41.157 |  | 39.09 |  |
| 17 | ˗1.41 | ˗1.09 | ˗1.71 | Phosphoglycerate mutase | A0A088A4K0 | 15387.60 | 7.80/33.107 | 9.36/ 35.361 | 74.36 | bisphosphoglycerate mutase activity; phosphoglycerate mutase activity |
| 45 | – | ˗1.19 | ˗1.38 |  |  | 303.42 | 4.45/36.630 |  | 15.38 |  |
| 21 | ˗1.80 | ˗1.73 | ˗2.22 | Inorganic pyrophosphatase | A0A088ANF1 | 22059.11 | 5.50/37.782 | 6.57/37.661 | 76.2 | inorganic diphosphatase activity; magnesium ion binding |
| 23 | ˗2.48 | ˗2.52 | ˗2.93 | Arginine kinase | A0A088ARZ8 | 963.48 | 6.06/38.923 | 6.11/43.411 | 38.38 | ATP binding; kinase activity |
| 24 | ˗2.40 | ˗1.45 | ˗2.50 | Malate dehydrogenase | A0A087ZYQ1 | 7850.33 | 6.77/39.214 | 6.25/ 36.159 | 49.55 | L˗malate dehydrogenase activity |
| 25 | ˗1.92 | ˗1.26 | ˗1.95 |  |  | 1175.97 | 7.42/39.670 |  | 32.13 |  |
| 30 | ˗1.51 | ˗1.32 | ˗2.02 | Aldose 1˗epimerase | A0A087ZNJ7 | 200.41 | 6.66/46.632 | 5.95/42.255 | 5.97 | aldose 1˗epimerase activity; carbohydrate binding |
| 33 | ˗1.60 | ˗1.85 | ˗2.47 | Glucose˗6˗phosphate isomerase | A0A087ZPV4 | 1730.46 | 8.46/63.974 | 7.73/62.967 | 54.94 | glucose˗6˗phosphate isomerase activity |
| 53 | Ø | Ø | Ø | Phosphomannomutase | A0A088A245 | 1380.67 | 6.29/30.120 | 5.68/30.390 | 60.31 | phosphomannomutase activity |
| 20 | ˗1.11 | ˗1.21 | ˗1.41 | Transaldolase | A0A087ZPV0 | 3804.99 | 6.68/36.948 | 8.12/ 37.178 | 53.92 | sedoheptulose˗7˗phosphate:D˗glyceraldehyde˗3˗phosphate glyceronetransferase activity |
| 23 | ˗2.48 | ˗2.52 | ˗2.93 |  |  | 1086.21 | 6.06/38.923 |  | 42.17 |  |
| 24 | ˗2.40 | ˗1.45 | ˗2.50 |  |  | 1085.61 | 6.77/39.214 |  | 40.96 |  |
| 58 | Ø | ˗1.20 | Ø |  |  | 120.95 | 8.19/37.224 |  | 16.87 |  |
| **Antioxidant system** | | | | | | | | | | |
| 11 | ˗1.43 | ˗1.63 | ˗2.05 | Superoxide dismutase [Cu˗Zn] | A0A088A933 | 11015.95 | 7.12/17.376 | 6.21/15.634 | 92.76 | metal ion binding; superoxide dismutase activity |
| 50 | ˗1.07 | – | Ø |  |  | 5280.47 | 6.93/17.789 |  | 58.55 |  |
| 14 | ˗1.47 | ˗1.21 | ˗1.61 | Peroxiredoxin 1 | A0A087ZND2 | 742.39 | 7.41/25.657 | 5.65/ 21.787 | 31.96 | peroxiredoxin activity |
| 41 | – | ˗1.68 | ˗2.19 |  |  | 24167.89 | 6.30/22.151 |  | 81.44 |  |
| 16 | ˗2.46 | ˗1.58 | ˗2.65 | Pyridoxamine 5 ˗phosphate oxidase | A0A088ASV9 | 723.35 | 8.68/28.711 | 8.41/30.041 | 31.23 | FMN binding; pyridoxamine˗phosphate oxidase activity |
| 16 | ˗2.46 | ˗1.58 | ˗2.65 | 15˗hydroxyprostaglandin dehydrogenase [NAD(+)]˗like | A0A088A500 | 223.44 | 8.68/28.711 | 8.56/28.776 | 44.49 | alcohol dehydrogenase (NAD) activity |
| 20 | ˗1.11 | ˗1.21 | ˗1.41 | Lambda˗crystallin homolog | A0A087ZPK0 | 85.63 | 6.68/36.948 | 6.56/35.861 | 14.2 | 3˗hydroxyacyl˗CoA dehydrogenase activity |
| 25 | ˗1.92 | ˗1.26 | ˗1.95 |  |  | 12355.92 | 7.42/39.670 |  | 75.39 |  |
| 20 | ˗1.11 | ˗1.21 | ˗1.41 | Trans˗1,2˗dihydrobenzene˗1,2˗diol dehydrogenase˗like | H9K9P8 | 2652.83 | 6.68/36.948 | 6.07/36.899 | 52.57 | oxidoreductase activity |
| 22 | ˗1.55 | ˗1.60 | ˗2.20 | Glycerol˗3˗phosphate dehydrogenase [NAD(+)] | A0A087ZRH2 | 12268.41 | 6.97/37.929 | 5.39/ 31.172 | 89.74 | glycerol˗3˗phosphate dehydrogenase [NAD+] activity; NAD binding |
| 27 | ˗1.18 | ˗1.20 | ˗1.45 |  |  | 2700.72 | 6.92/42.758 |  | 58.95 |  |
| 47 | – | ˗1.08 | ˗1.51 |  |  | 257.85 | 6.93/85.626 |  | 20.70 |  |
| 24 | ˗2.40 | ˗1.45 | ˗2.50 | Malate dehydrogenase | A0A087ZYQ1 | 7850.33 | 6.77/39.214 | 6.25/ 36.159 | 49.55 | L˗malate dehydrogenase activity |
| 25 | ˗1.92 | ˗1.26 | ˗1.95 |  |  | 1175.97 | 7.42/39.670 |  | 32.13 |  |
| 25 | ˗1.92 | ˗1.26 | ˗1.95 | Aldose reductase˗like | A0A088AGI8 | 10563.21 | 7.42/39.670 | 6.26/36.082 | 82.08 | oxidoreductase activity |
| 27 | ˗1.18 | ˗1.20 | ˗1.45 | Glycerol˗3˗phosphate dehydrogenase [NAD(+)] | A0A087ZRH1 | 3882.57 | 6.92/42.758 | 9.21/8.777 | 56.41 | glycerol˗3˗phosphate dehydrogenase [NAD+] activity; NAD binding |
| 29 | ˗1.28 | ˗2.29 | ˗2.57 | Probable medium˗chain specific acyl˗ mitochondrial | A0A087ZTI9 | 7524.57 | 7.29/45.000 | 8.51/46.520 | 54.44 | acyl˗CoA dehydrogenase activity; flavin adenine dinucleotide binding |
| 35 | ˗1.11 | – | ˗1.36 | Glutathione S˗transferase S1 | C3VMN1 | 2591.21 | 9.66/29.021 | 8.77/23.762 | 39.22 | transferase activity |
| 35 | ˗1.11 | – | ˗1.36 | 3˗hydroxyacyl˗ dehydrogenase type˗2 | A0A088AU44 | 3388.52 | 9.66/29.021 | 9.07/27.099 | 47.24 | oxidoreductase activity |
| 43 | – | ˗1.04 | ˗1.13 | 15˗hydroxyprostaglandin dehydrogenase [NAD(+)]˗like | A0A088A4Z9 | 292.27 | 5.61/26.585 | 5.04/28.332 | 25.76 | alcohol dehydrogenase (NAD) activity |
| 48 | – | ˗1.15 | ˗1.21 | Rab s geranylgeranyltransferase component A1 | A0A087ZUJ9 | 3474.99 | 4.90/88.937 | 4.72/57.901 | 39.33 | GDP˗dissociation inhibitor activity; oxidoreductase activity |
| 43 | – | ˗1.04 | ˗1.13 | Glutathione peroxidase | A0A088AB47 | 219.72 | 5.61/26.585 | 5.64/ 23.133 | 27.36 | glutathione peroxidase activity |
| 43 | – | ˗1.04 | ˗1.13 | Thioredoxin domain˗containing 9 | A0A087ZT30 | 125.50 | 5.61/26.585 | 6.02/25.134 | 15.81 | cell redox homeostasis |
| 42 | – | ˗1.31 | ˗1.49 | Farnesol dehydrogenase˗like | H9K5X4 | 26617.52 | 5.01/26.552 | 4.92/26.332 | 48.78 | oxidoreductase activity |
| 43 | – | ˗1.04 | ˗1.13 |  |  | 1067.38 | 5.61/26.585 |  | 47.56 |  |
| 46 | – | ˗1.60 | ˗1.63 | Prophenoloxidase | Q86MV4 | 128.91 | 6.99/78.43 | 6.28/80.094 | 10.10 | metal ion binding; oxidoreductase activity |
| **Biosynthesis processes** | | | | | | | | | | |
| 12 | ˗1.00 | ˗1.01 | ˗1.15 | Prostaglandin E synthase 3 | A0A088ARS8 | 326.25 | 4.90/18.909 | 4.32/20.122 | 18.75 | – |
| 16 | ˗2.46 | ˗1.58 | ˗2.65 | Pyridoxamine 5 ˗phosphate oxidase | A0A088ASV9 | 723.35 | 8.68/28.711 | 8.41/30.041 | 31.23 | FMN binding; pyridoxamine˗phosphate oxidase activity |
| 27 | ˗1.18 | ˗1.20 | ˗1.45 | Glutamine synthetase 2 cytoplasmic isoform X1 | A0A088A2A8 | 20617.08 | 6.92/42.758 | 8.37/45.361 | 58.27 | glutamate˗ammonia ligase activity |
| 30 | ˗1.51 | ˗1.32 | ˗2.02 |  |  | 3636.45 | 6.66/46.632 |  | 41.48 |  |
| 27 | ˗1.18 | ˗1.20 | ˗1.45 | Glutamine synthetase 2 cytoplasmic | A0A088AIM3 | 18685.47 | 6.92/42.758 | 5.76/34.560 | 58.39 | glutamate˗ammonia ligase activity |
| 30 | ˗1.51 | ˗1.32 | ˗2.02 |  |  | 2737.79 | 6.66/46.632 |  | 39.03 |  |
| 40 | – | ˗1.38 | ˗1.92 | 6˗pyruvoyl tetrahydrobiopterin synthase | A0A088AJ91 | 292.88 | 7.54/15.414 | 6.96/ 16.660 | 33.1 | 6˗pyruvoyltetrahydropterin synthase activity; tetrahydrobiopterin biosynthetic process |
| 49 | – | ˗1.07 | ˗2.05 | Alanine aminotransferase 1 | A0A087ZNZ2 | 300.66 | 8.26/59.726 | 8.53/60.829 | 21.55 | catalytic activity; pyridoxal phosphate binding; biosynthetic process |
| 56 | Ø | ˗1.00 | Ø | Hydroxyacylglutathione mitochondrial isoform X1 | A0A087ZZF5 | 1908.96 | 6.47/33.170 | 6.83/34.129 | 38.93 | hydroxyacylglutathione hydrolase activity |
| **Amino acid metabolism** | | | | | | | | | | |
| 15 | ˗1.68 | ˗1.78 | ˗2.19 | Protein˗L˗isoaspartate O˗methyltransferase | A0A087ZYZ9 | 941.70 | 5.39/26.334 | 5.17/28.618 | 42.80 | protein˗L˗isoaspartate (D˗aspartate) O˗methyltransferase activity |
| 17 | ˗1.41 | ˗1.09 | ˗1.71 | S˗methyl˗5'˗thioadenosine phosphorylase | A0A088A605 | 1699.47 | 7.80/33.107 | 6.75/ 31.553 | 40.00 | S˗methyl˗5˗thioadenosine phosphorylase activity |
| 27 | ˗1.18 | ˗1.20 | ˗1.45 | Glutamine synthetase 2 cytoplasmic isoform X1 | A0A088A2A8 | 20617.08 | 6.92/42.758 | 8.37/45.361 | 58.27 | glutamate˗ammonia ligase activity |
| 30 | ˗1.51 | ˗1.32 | ˗2.02 |  |  | 3636.45 | 6.66/46.632 |  | 41.48 |  |
| 27 | ˗1.18 | ˗1.20 | ˗1.45 | Glutamine synthetase 2 cytoplasmic | A0A088AIM3 | 18685.47 | 6.92/42.758 | 5.76/34.560 | 58.39 | glutamate˗ammonia ligase activity |
| 30 | ˗1.51 | ˗1.32 | ˗2.02 |  |  | 2737.79 | 6.66/46.632 |  | 39.03 |  |
| 49 | – | ˗1.07 | ˗2.05 | Alanine aminotransferase 1 | A0A087ZNZ2 | 300.66 | 8.26/59.726 | 8.53/60.829 | 21.55 | catalytic activity; pyridoxal phosphate binding; biosynthetic process |
| **Transcription/translation** | | | | | | | | | | |
| 07 | ˗1.22 | ˗1.11 | ˗1.53 | Ubiquitin˗60S ribosomal L40 | A0A088AN20 | 986.55 | 7.39/11.386 | 9.87/14.703 | 21.88 | structural constituent of ribosome |
| 25 | ˗1.92 | ˗1.26 | ˗1.95 | Eukaryotic translation initiation factor 3 subunit I | A0A088A549 | 1180.44 | 7.42/39.670 | 6.31/36.627 | 24.54 | translation initiation factor activity |
| 31 | ˗1.03 | ˗1.07 | ˗1.74 | Elongation factor 1˗gamma | A0A088A224 | 4843.86 | 5.56/49.125 | 6.01/48.986 | 52.24 | translation elongation factor activity |
| 32 | ˗1.33 | ˗1.08 | ˗1.73 | Tyrosine˗˗tRNA ligase | A0A088AMC1 | 5497.27 | 6.54/57.427 | 5.73/58.752 | 62.45 | ATP binding; tRNA binding; tyrosine˗tRNA ligase activity |
| 51 | – | ˗1.24 | Ø | U3 small nucleolar RNA˗associated 4 homolog A | A0A088ABI2 | 178.17 | 5.74/116.750 | 9.31/25.315 | 5.24 | rRNA processing |
| 60 | – | – | Ø | Eukaryotic initiation factor 4A˗I | A0A088AJJ6 | 11788.26 | 5.92/56.223 | 5.29/48.156 | 62.17 | ATP binding; helicase activity; nucleic acid binding |
| **Protein folding/binding** | | | | | | | | | | |
| 02 | Ø | Ø | Ø | Hsp70˗binding 1 | A0A087ZNK4 | 537.28 | 4.63/41.157 | 4.87/42.752 | 29.37 | – |
| 08 | ˗2.04 | ˗2.10 | ˗2.41 | 10 kda heat shock mitochondrial | A0A088ARQ3 | 13544.57 | 8.83/13.515 | 8.01/ 11.403 | 81.73 | ATP binding; chaperone |
| 09 | ˗1.33 | ˗1.57 | ˗1.68 | Profilin | A0A087ZNT2 | 9253.49 | 5.68/13.707 | 5.64/13.803 | 40.48 | actin binding |
|  |  |  |  | Profilin | Q6QEJ7 | 9253.49 |  | 5.64/13.720 | 40.48 | actin binding |
|  |  |  |  | Profilin | A0A087ZYF1 | 5384.47 |  | 5.73/9.694 | 21.35 | actin binding |
| 32 | ˗1.33 | ˗1.08 | ˗1.73 | T˗complex 1 subunit eta | A0A087ZUG4 | 3980.18 | 6.54/57.427 | 6.03/59.935 | 60.15 | ATP binding; unfolded protein binding |
| 34 | ˗1.15 | ˗1.14 | ˗1.70 | 60 kda heat shock mitochondrial˗like | A0A088ART2 | 44137.55 | 5.60/66.472 | 5.64/60.413 | 81.05 | ATP binding |
| **Olfactory system** | | | | | | | | | | |
| 10 | ˗1,82 | ˗2,16 | ˗2,17 | Odorant binding 14 | A0A088A4K9 | 18513.07 | 5.84/15.597 | 5.64/ 14.883 | 80.00 | odorant binding |
| 12 | ˗1.00 | ˗1.01 | ˗1.15 |  |  | 118.03 | 4.90/18.909 |  | 11.54 |  |
| **Learning and memory** | | | | | | | | | | |
| 44 | – | ˗1.17 | ˗1.46 | 14˗3˗3 epsilon | A0A087ZUP0 | 1893.91 | 4.74/29.021 | 4.78/29.111 | 51.17 | – |
| 44 | – | ˗1.17 | ˗1.46 | 14˗3˗3 zeta | A0A088AHG5 | 9829.84 | 4.74/29.021 | 5.41/45.145 | 47.86 | protein domain specific binding |
| 57 | Ø | ˗1.00 | Ø |  |  | 1423.78 | 6.26/34.299 |  | 27.71 |  |
| **Other/unknown functions** | | | | | | | | | | |
| 11 | ˗1.43 | ˗1.63 | ˗2.05 | Nucleoside diphosphate kinase | A0A088ATV8 | 30733.69 | 7.12/17.376 | 6.75/17.642 | 89.61 | ATP binding; nucleoside diphosphate kinase activity |
| 12 | ˗1.00 | ˗1.01 | ˗1.15 | Calexcitin˗2 | A0A088AE24 | 815.55 | 4.90/18.909 | 4.79/21.347 | 19.67 | calcium ion binding |
| 13 | ˗1.29 | ˗1.66 | ˗2.19 | D2˗like isoform X1 | A0A088AEV7 | 5642.22 | 9.64/23.171 | 9.18/23.645 | 31.1 | – |
| 13 | ˗1.29 | ˗1.66 | ˗2.19 | Proteasome subunit beta type | A0A087ZZ62 | 356.54 | 9.64/23.171 | 7.08/31.653 | 16.67 | Threonine˗type endopeptidase activity |
| 14 | ˗1.47 | ˗1.21 | ˗1.61 | GTP˗binding nuclear protein | A0A087ZWI7 | 3163.30 | 7.41/25.657 | 6.96/24.644 | 32.09 | GTPase activity; GTP binding |
| 15 | ˗1.68 | ˗1.78 | ˗2.19 | Rho GDP˗dissociation inhibitor 2 isoform X1 | A0A0B4J2M0 | 8295.28 | 5.39/26.334 | 5.18/23.522 | 69.27 | Rho GDP˗dissociation inhibitor activity |
| 18 | ˗1.74 | ˗1.74 | ˗2.00 | Phytanoyl˗ dioxygenase domain˗containing 1 homolog | A0A088A5Q3 | 1953.86 | 7.12/33.170 | 6.46/32.676 | 59.22 | – |
| 18 | ˗1.74 | ˗1.74 | ˗2.00 | Alpha˗tocopherol transfer ˗like | A0A088AC27 | 3510.15 | 7.12/33.170 | 6.41/32.378 | 64.52 | transporter activity |
| 18 | ˗1.74 | ˗1.74 | ˗2.00 | Phosphatidylinositol transfer alpha isoform | A0A088AUP1 | 17188.21 | 7.12/33.170 | 6.20/31.549 | 88.19 | transporter activity |
| 52 | Ø | – | Ø |  |  | 276.62 | 6.12/28.952 |  | 16.24 |  |
| 18 | ˗1.74 | ˗1.74 | ˗2.00 | Electron transfer flavo subunit mitochondrial | A0A088A872 | 12682.52 | 7.12/33.170 | 8.49/35.379 | 67.57 | electron carrier activity; flavin adenine dinucleotide binding |
| 19 | ˗1.16 | ˗1.43 | ˗1.63 | 26S proteasome non˗atpase regulatory subunit 14 | A0A087ZR05 | 5199.85 | 6.12/34.233 | 5.87/34.616 | 60.45 | – |
| 19 | ˗1.16 | ˗1.43 | ˗1.63 | Glyoxalase domain˗containing 4 | A0A088AM27 | 19021.87 | 6.12/34.233 | 5.51/32.128 | 71.89 | – |
| 21 | ˗1.80 | ˗1.73 | ˗2.22 | Serine/threonine˗protein phosphatase | A0A088A299 | 364.29 | 5.50/37.782 | 5.30/ 35.443 | 37.54 | phosphoprotein phosphatase activity |
| 22 | ˗1.55 | ˗1.60 | ˗2.20 | Guanine nucleotide˗binding subunit beta˗2 | A0A087ZSH2 | 1137.35 | 6.97/37.929 | 6.15/38.094 | 38.73 | signal transduction |
| 24 | ˗2.40 | ˗1.45 | ˗2.50 | Probable tRNA N6˗adenosine threonylcarbamoyltransferase | A0A0B4J2M8 | 549.30 | 6.77/39.214 | 6.09/ 37.330 | 37.31 | metal ion binding; N(6)˗L˗threonylcarbamoyladenine synthase |
| 26 | ˗1.26 | ˗1.85 | ˗2.27 | Not identified | – | – | 5.84/40.766 | – | – | – |
| 31 | ˗1.03 | ˗1.07 | ˗1.74 | Phosphotransferase | A0A088A6W8 | 1994.27 | 5.56/49.125 | 5.87/49.643 | 32.36 | ATP binding; glucose binding; hexokinase activity |
| 35 | ˗1.11 | – | ˗1.36 | Isochorismatase domain˗containing 2˗like | A0A088AA38 | 2661.54 | 9.66/29.021 | 8.64/ 22.439 | 63.05 | catalytic activity |
| 36 | ˗1.09 | – | ˗1.56 | Enolase isoform X2 | A0A088AST9 | 33993.14 | 6.95/56.980 | 6.54/ 39.789 | 60.93 | magnesium ion binding; phosphopyruvate hydratase activity |
| 37 | ˗1.11 | – | Ø | Tubulin beta chain | A0A087ZYZ1 | 1983.68 | 4.75/57.746 | 4.75/50.175 | 50.56 | GTPase activity; GTP binding; structural constituent of cytoskeleton |
| 38 | ˗1.76 | – | ˗2.27 | Transferrin | A0A088AFH7 | 11329.54 | 7.33/79.621 | 6.77/78.629 | 75.14 | metal ion binding; iron ion homeostasis |
| 39 | ˗1.15 | – | ˗1.35 | Glucosidase 2 subunit beta | A0A088AUN3 | 136.54 | 4.24/80.012 | 4.44/59.829 | 10.27 | calcium ion binding |
| 40 | – | ˗1.38 | ˗1.92 | Probable fatty acid˗binding isoform X2 | A0A088AE74 | 21838.05 | 7.54/15.414 | 8.47/15.223 | 66.17 | lipid binding; transporter activity |
| 40 | – | ˗1.38 | ˗1.92 | Ubiquitin˗conjugating enzyme E2˗17 kda | A0A088AG51 | 426.32 | 7.54/15.414 | 6.81/ 16.678 | 23.81 | ATP binding |
| 40 | – | ˗1.38 | ˗1.92 | Histidine triad nucleotide˗binding 1 | A0A088AA27 | 549.33 | 7.54/15.414 | 7.05/ 13.973 | 18.25 | catalytic activity |
| 43 | – | ˗1.04 | ˗1.13 | Alpha˗crystallin A chain isoform X2 | A0A088APK0 | 4497.58 | 5.61/26.585 | 5.36/7.684 | 70.77 | – |
| 45 | – | ˗1.19 | ˗1.38 | Annexin | A0A088AJH1 | 87.12 | 4.45/36.630 | 4.60/ 35.882 | 5.57 | Calcium˗dependent phospholipid binding; calcium ion binding |
| 51 | – | ˗1.24 | Ø | Ubiquitin˗like modifier˗activating enzyme 1 | A0A088AVS9 | 70.58 | 5.74/116.750 | 5.41/117.106 | 5.58 | ATP binding; small protein activating enzyme activity |
| 52 | Ø | – | Ø | Hydroxypyruvate isomerase | A0A088A893 | 2621.24 | 6.12/28.952 | 5.93/17.996 | 57.69 | – |
| 54 | Ø | – | Ø | Pyridoxal kinase | A0A088AHV4 | 4901.56 | 6.36/34.684 | 6.11/33.348 | 42.91 | pyridoxal kinase activity |
| 59 | – | – | ˗1.31 | Electron transfer flavo subunit beta | A0A088A7F1 | 2484.85 | 7.59/29.437 | 6.46/27.581 | 69.96 | electron carrier activity |

**Supplementary Table S2.** Identification of upregulated protein spots (ANOVA; α= 0.05) in nurse honeybees exposed to pyraclostrobin (P), fipronil (F) and pyraclostrobin + fipronil (PF). The expression analysis was performed with Image Master Platinum software, using as a reference the proteome of bees not exposed to pesticides (control), and the protein identification was performed by ESI-MS/MS. A positive signal (+) indicated potential increase in protein expression in bees exposed to pesticides. ^a^Spot ID numbers refer to the numbers on the 2D-PAGE gels shown in Fig. 1. ^b,c^Protein accession number and score according to the MASCOT software. ^d^Experimental pI and Mw of proteins obtained in Platinum Image Master software from gel analyses. ^e^Theoretical pI and Mw of proteins obtained in UniProtKB/Swiss-Prot database. ^f^Protein coverage indicates the percentage of residues from the sequences that were identified by the spectrometer for each protein. ^g^Role in biological process according UniProtKB-KW database.

| Spot ID^a^ | Fold  Change in P | Fold  Change in F | Fold  Change in PF | Protein | Access^b^ | Score^c^ | pI/MM experimental^d^ | pI/MM theoretical^e^ | Coverage (%)^f^ | Molecular function^g^ |
| --- | --- | --- | --- | --- | --- | --- | --- | --- | --- | --- |
| **Stress response** | | | | | | | | | | |
| 63 | +1.58 | +2.27 | +2.51 | Heat shock 70 kda cognate 5 | A0A087ZTY7 | 72.73 | 7.58/74.873 | 6.38/75.404 | 11.21 | ATP binding; unfolded protein binding |
| 70 | +1.03 | – | +1.18 |  |  | 62.73 | 7.41/82.328 |  | 10.34 |  |
| 64 | +1.19 | +1.13 | +1.72 | Heat shock 70 kda 4 isoform X1 | A0A088AGW8 | 3567.68 | 6.19/97.918 | 5.74/ 7.717 | 54.90 | ATP binding |
| 74 | – | – | +1.45 |  |  | 404.45 | 5.98/108.840 |  | 7.90 |  |
| 69 | +1.10 | – | +1.41 | Heat shock 70 kda cognate 3 | A0A088ACF4 | 91.66 | 5.78/68.396 | 5.21/72.478 | 14.2 | ATP binding |
| **Carbohydrate metabolism and energy synthesis** | | | | | | | | | | |
| 63 | +1.58 | +2.27 | +2.51 | Malic enzyme | A0A088AES7 | 117.73 | 7.58/74.873 | 7.55/69.763 | 15.20 | malate dehydrogenase (decarboxylating) (NAD+) activity; metal ion binding; NAD binding |
| 67 | +1.10 | – | +1.70 | Pyruvate dehydrogenase E1 component subunit mitochondrial | A0A088AUU5 | 16078.73 | 5.64/35.073 | 5.29/36.322 | 59.7 | pyruvate dehydrogenase (acetyl˗transferring) activity |
| 72 | – | – | +1.36 | Pyruvate kinase | A0A088A5I8 | 863.14 | 7.68/61.863 | 6.92/66.565 | 49.00 | kinase activity; magnesium ion binding; potassium ion binding; pyruvate kinase activity |
| **Transcription/translation** | | | | | | | | | | |
| 66 | +1.05 | – | +1.05 | Eukaryotic translation initiation factor 3 subunit K | A0A088ATI4 | 176.62 | 5.84/24.009 | 5.52/24.860 | 31.02 | ribosome binding; translation initiation factor activity |
| **Protein folding/binding** | | | | | | | | | | |
| 62 | +1.04 | +1.09 | +1.14 | Lethal (2) essential for life˗like | A0A088A3R7 | 5098.71 | 5.65/22.952 | 5.35/ 22.462 | 63.21 | – |
| 63 | +1.58 | +2.27 | +2.51 | Heat shock 70 kda cognate 5 | A0A087ZTY7 | 72.73 | 7.58/74.873 | 6.38/75.404 | 11.21 | ATP binding; unfolded protein binding |
| 70 | +1.03 | – | +1.18 |  |  | 72.73 | 7.41/82.328 |  | 10.34 |  |
| 64 | +1.19 | +1.13 | +1.72 | Heat shock 70 kda 4 isoform X1 | A0A088AGW8 | 3567.68 | 6.19/97.918 | 5.74/ 7.717 | 54.90 | ATP binding |
| 74 | – | – | +1.45 |  |  | 404.45 | 5.98/108.840 |  | 7.90 |  |
| 66 | +1.05 | – | +1.05 | Lethal (2) essential for life˗like | A0A088A3R9 | 200.63 | 5.84/24.009 | 5.40/ 23.002 | 22.96 | – |
| 69 | +1.10 | – | +1.41 | Heat shock 70 kda cognate 3 | A0A088ACF4 | 91.66 | 5.78/68.396 | 5.21/72.478 | 14.2 | ATP binding |
| **Other / unknown functions** | | | | | | | | | | |
| 01 | +1.22 | +1.37 | +1.52 | Not identified | – | – | 4.51/9.663 | – | – | – |
| 61 | +2.53 | +2.18 | +3.17 | Not identified | – | – | 4.55/10.341 | – | – | – |
| 62 | +1.04 | +1.09 | +1.14 | Lactoylglutathione lyase | A0A088ALN9 | 536.31 | 5.65/22.952 | 5.45/ 21.100 | 20.22 | lactoylglutathione lyase activity; metal ion binding |
| 65 | – | +1.14 | +2.14 | Translationally˗controlled tumor homolog | A0A087ZVC8 | 3042.71 | 4.53/21.327 | 4.57/ 19.843 | 46.51 | – |
| 67 | +1.10 | – | +1.70 | F˗actin˗capping subunit alpha | A0A088A627 | 3391.82 | 5.64/35.073 | 5.15/34.139 | 57.33 | phosphatase activity |
| 68 | +1.01 | – | +1.30 | 26S proteasome non˗atpase regulatory subunit 13 | A0A088AD16 | 2878.38 | 6.54/42.904 | 6.29/42.315 | 42.09 | – |
| 69 | +1.10 | – | +1.41 | Protein disulfide˗isomerase | A0A088A7Y2 | 8979.38 | 5.78/68.396 | 5.57/55.857 | 58.78 | protein disulfide isomerase activity |
| 69 | +1.10 | – | +1.41 | Calpain˗A isoform X3 | A0A087ZX44 | 164.52 | 5.78/68.396 | 4.80/88.160 | 15.57 | calcium˗dependent cysteine˗type endopeptidase activity; calcium ion binding |
| 71 | – | – | +1.13 | Not identified | – | – | 4.07/25.840 | – | – | – |
| 72 | – | – | +1.36 | Serine˗˗trna cytoplasmic | A0A088A397 | 3042.41 | 7.68/61.863 | 6.82/57.414 | 38.91 | ATP binding; serine˗tRNA ligase activity |
| 73 | – | – | +1.05 | Carboxylic ester hydrolase | A0A087ZXF6 | 32765.91 | 5.90/71.385 | 5.56/59.668 | 63.38 | hydrolase activity |
| 73 | – | – | +1.05 | Dihydropyrimidinase isoform X2 | A0A088ALH3 | 349.01 | 5.90/71.385 | 5.84/ 74.941 | 25.58 | hydrolase activity, acting on carbon˗nitrogen (but not peptide) bonds |
